# A Brain-Derived Neurotrophic Factor-Based p75^NTR^ Peptide Mimetic Ameliorates Experimental Autoimmune Neuritis Induced Axonal Pathology and Demyelination

**DOI:** 10.1523/ENEURO.0142-17.2017

**Published:** 2017-07-04

**Authors:** David G. Gonsalvez, Giang Tran, Jessica L. Fletcher, Richard A. Hughes, Suzanne Hodgkinson, Rhiannon J. Wood, Sang Won Yoo, Mithraka De Silva, Wong W. Agnes, Catriona McLean, Paul Kennedy, Trevor J. Kilpatrick, Simon S. Murray, Junhua Xiao

**Affiliations:** 1 Department of Anatomy and Neuroscience, The University of Melbourne, VIC 3010, Australia; 2 Department of Pharmacology and Therapeutics, The University of Melbourne, VIC 3010, Australia; 3 Liverpool Hospital, The University of New South Wales, NSW 2170, Australia; 4 Victorian Neuromuscular Laboratory Services, Alfred Health, VIC 3004, Australia

**Keywords:** axonal damage, BDNF, demyelination, experimental autoimmune neuritis, p75NTR

## Abstract

Axonal damage and demyelination are major determinants of disability in patients with peripheral demyelinating neuropathies. The neurotrophin family of growth factors are essential for the normal development and myelination of the peripheral nervous system (PNS), and as such are potential therapeutic candidates for ameliorating axonal and myelin damage. In particular, BDNF promotes peripheral nerve myelination via p75 neurotrophin receptor (p75^NTR^) receptors. Here, we investigated the therapeutic efficacy of a small structural mimetic of the region of BDNF that binds to p75^NTR^ (cyclo-dPAKKR) in experimental autoimmune neuritis (EAN), an established animal model of peripheral demyelinating neuropathy. Examination of rodents induced with EAN revealed that p75^NTR^ is abundantly expressed in affected peripheral nerves. We found that systemic administration of cyclo-dPAKKR ameliorates EAN disease severity and accelerates recovery. Animals treated with cyclo-dPAKKR displayed significantly better motor performance compared to control animals. Histological assessment revealed that cyclo-dPAKKR administration limits the extent of inflammatory demyelination and axonal damage, and protects against the disruption of nodal architecture in affected peripheral nerves. In contrast, a structural control peptide of cyclo-dPAKKR exerted no influence. Moreover, all the beneficial effects of cyclo-dPAKKR in EAN are abrogated in p75^NTR^ heterozygous mice, strongly suggesting a p75^NTR^-dependent effect. Taken together, our data demonstrate that cyclo-dPAKKR ameliorates functional and pathological defects of EAN in a p75^NTR^-dependant manner, suggesting that p75^NTR^ is a therapeutic target to consider for future treatment of peripheral demyelinating diseases and targeting of p75^NTR^ is a strategy worthy of further investigation.

## Significance Statement

This study demonstrates that a BDNF-based peptide mimetic ameliorates the clinical and functional deficits in experimental autoimmune neuritis (EAN), an animal model of peripheral demyelinating neuropathy. Concordant with these findings, treatment significantly reduced immune cell infiltration, the extent of peripheral demyelination and axonal pathology. Genetic analyses identified these effects were mediated through p75 neurotrophin receptor (p75^NTR^). This study identifies a novel avenue for myelin and axonal protection in peripheral demyelinating neuropathy through targeting p75^NTR^; however, additional studies are required to identify the precise cellular mechanism underlying these effects. The strong protective effect that the mimetic exerts on axonal damage paves the way for studying its application in other peripheral neuropathies in the future.

## Introduction

Demyelination and axonal degeneration are pathologic hallmarks of injured peripheral nerve in acquired demyelinating peripheral neuropathies (Hughes et al., 2007). However, current therapies for these diseases are anti-inflammatory and do not prevent neurologic symptoms underpinned by persistent demyelination and secondary axonal degeneration (Hughes et al., 2007), demonstrating a need for novel neuroprotective therapies that can be administered as adjuncts to existing immunomodulatory therapies for the treatment of these diseases.

The neurotrophins are a family of soluble growth factors essential for peripheral nervous system (PNS) development and myelination (Chan et al., 2001; Cosgaya et al., 2002; Xiao et al., 2009a). They comprise nerve growth factor (NGF), BDNF, neurotrophin (NT)-3, and NT-4/5. Their biological effects are mediated via two distinct classes of receptors: the tropomyosin-related kinase (Trk) receptors and the p75 neurotrophin receptor (p75^NTR^; Kaplan and Miller, 2000). Different neurotrophins exert distinct influences on peripheral myelination depending on the cognate receptor expressed by peripheral neurons (Xiao et al., 2009a). We and others have demonstrated that BDNF promotes myelination via acting on p75^NTR^ (Cosgaya et al., 2002; Xiao et al., 2009b), whereas it inhibits myelination via activating TrkB (Xiao et al., 2009b). Moreover, analyses of mutant mice indicate that p75^NTR^ is required for the survival of a subset of peripheral neurons (Lee et al., 1992). In this context, selective targeting of p75^NTR^ could be an approach that, in principle, exerts a beneficial effect on myelin repair and protection against axonal damage after peripheral nerve injury.

This has led us to develop the cyclic pentapeptide cyclo-[dPro-Ala-Lys-Lys-Arg] (cyclo-dPAKKR), a structural mimetic of BDNF (Fletcher et al., 2008). Cyclo-dPAKKR is presented in a highly conformationally constrained cyclic template that contains the positively charged ‘Lys-Lys-Arg’ tri-peptide motif required for BDNF to bind to p75^NTR^ (Rydén et al., 1995). Importantly, cyclo-dPAKKR contains no sequence similarity to the loop 2 region of BDNF that binds TrkB and does not cause TrkB autophosphorylation (Fletcher et al., 2008). Equally, it contains no sequence similarity to the N-terminal portion of pro-BDNF that binds the sortilin-p75^NTR^ receptor complex and does not induce the death of sensory neurons or Schwann cells in culture (Fletcher et al., 2008). Cyclo-dPAKKR displays drug-like properties, in that it is of low molecular weight (relative molecular mass of 580), metabolically stable in rodent plasma *in vitro* and readily crosses cell membranes (Fletcher et al., 2008; Fletcher and Hughes, 2009). Collectively, these properties of cyclo-dPAKKR render it a unique pharmacological tool to investigate the selective influence of BDNF on p75^NTR^, without the concomitant activation of TrkB or the sortilin-p75^NTR^ proneurotrophin receptor complex. Indeed, we have previously shown that cyclo-dPAKKR promotes the survival of peripheral neurons *in vitro* (Fletcher et al., 2008) and peripheral nerve myelination *in vitro* and *in vivo*, an effect dependent on the expression of p75^NTR^ (Xiao et al., 2013).

In this study, we investigated the therapeutic potential of cyclo-dPAKKR in experimental autoimmune neuritis (EAN), a well-established model of demyelinating peripheral neuropathy. The disease course mirrors many of the morphologic and electrophysiological aspects of acute and chronic demyelinating peripheral neuropathy, given that on the one hand there is acute remission but that on the other hand there is incomplete recovery (Mäurer and Gold, 2002; Hughes and Cornblath, 2005; Yuki and Hartung, 2012). We found that p75^NTR^ is abundantly expressed in affected peripheral nerves in rodent EAN. Systemic administration of cyclo-dPAKKR ameliorates EAN disease course and motor deficits, and reduces the extent of demyelination and axonal damage, in a p75^NTR^-dependent manner. In contrast, a structural control peptide of cyclo-dPAKKR exerted no influence. Our data demonstrate that cyclo-dPAKKR exerts p75^NTR^-dependent neuroprotective effects in EAN, suggesting that targeting of p75^NTR^ is a strategy worthy of further investigation for the treatment of peripheral demyelinating diseases.

## Materials and Methods

### Animals

EAN disease was induced in Lewis rats (10–12 weeks old, female) bred and maintained at the Liverpool Hospital Animal Facility, New South Wales, and in C57B6 wild-type (WT, +/+) and NGFR/C57B6 (p75^NTR^ heterozygous, HET, +/−) mice housed at the Animal Facility of Florey Institute of Neuroscience and Mental Health Research, the University of Melbourne. The p75^NTR^ HET mice were obtained from a colony previously shown to be 95% congenic to the inbred C57B6 strain (Syroid et al., 2000), and subsequently back-crossed a further 12 generations. All animal procedures were approved by Animal Experimentation Ethics Committees at the University of New South Wales and University of Melbourne.

### Peptide syntheses and reagents

The conformationally-constrained cyclised active peptide cyclo-dPAKKR (dPro-Ala-Lys-Lys-Arg) and control peptide (Ala-dPro-Lys-Lys-Arg, cyclo-AdPKKR) were synthesised, purified and characterised as previously described (Fletcher et al., 2008; Xiao et al., 2013). All chemicals were obtained from Sigma-Aldrich unless otherwise indicated.

### EAN induction in rats

Adult Lewis rats were induced with EAN disease by inoculation with a peripheral myelin antigen using published methods (Hodgkinson et al., 1994; Tran et al., 2010, 2012). Briefly, 10- to 12-week-old female rats were immunized in the footpad with 200 μl of emulsion containing 4mg bovine peripheral nerve myelin and 1.5 mg of complete Freund's adjuvant prepared by emulsifying heat killed *Mycobacterium tuberculosis* (strain H37RA; Difco) in 100 μl saline and 100 μl incomplete Freund's adjuvant (Difco). Animals develop a severe disease initially consisting of paraplegia and eventually quadriparesis. Animals were monitored daily for weight loss, and clinical disease was scored as 0 for normal, 1 for limp tail, 2 for hind leg weakness, 3 for paraplegia, and 4 for quadriplegia.

### EAN induction in mice

Adult C57/B6 mice were induced with EAN disease as previously described (Gonsalvez et al., 2017). Briefly, six- to eight-week-old male mice (WT and p75 HET) were immunized twice (day 0 and day 8 after induction) by subcutaneous injection of myelin basic protein zero (P0) peptide 180–199 (P0_180–199_, sequence S-S-K-R-G-R-Q-T-P-V-L-Y-A-M-L-D-H-S-R-S), and 0.5 mg *Mycobacterium tuberculosis* (strain H 37 Ra; Difco 231141) emulsified in 25 μl saline and 25 μl of complete Freund’s adjuvant (Difco 263910 comprising; 3.75 μl of mannide monoolate + 21.25 μl of paraffin oil and 12.5 μg of desiccated killed and dried Myobacterium butyrcum). Mice received pertussis toxin (Ptx, Sigma) on day −1 (400 ng/mouse), and days 1 and 3 (300 ng/mouse) by intraperitoneal injection. All Ptx and inoculation injections were conducted on mice anesthetized by aerosol isoflurane 2% in normal air. Animals were monitored daily for weight loss, and clinical disease was scored as: 0 for normal, 1 for less lively, 2 for mild tail and hindlimb paresis, 3 for mild ataxia and limb paresis, 4 for severe ataxia and limb paresis and 5 for limb paralysis.

### Cyclo-dPAKKR treatment

EAN animals were intraperitoneally administrated with cyclo-dPAKKR (0.1–10 mg/kg/d) or vehicle control (PBS) daily after the day of disease induction (day 0). The doses of cyclo-dPAKKR were chosen based on its potency in promoting peripheral nerve myelination *in vivo* (Xiao et al., 2013). Rats were killed at day 17, the peak of clinical disease and when demyelination is maximal, or at day 24 when animals exhibit partial recovery (Tran et al., 2010, 2012). Cauda equina and sciatic nerves were dissected and prepared for Western blot, immunohistochemical, histologic, or electron microscopy (EM) analyses as described previously (Tran et al., 2002; Xiao et al., 2013). Six to seven rats per treatment group per time point were assessed. In some experiments, cyclo-dPAKKR (10 mg/kg/d), the control peptide (cyclo-AdPKKR, 10 mg/kg/d) or a vehicle (PBS) control was intraperitoneally administered to mice (p75^NTR^ HET and WT littermate controls) via mini osmotic pump (ALZET, CA pump#: 2006). Mice sciatic nerves were collected at day 23 (disease peak in murine EAN) and six mice per genotype per treatment group were assessed.

### Quantitative PCR (qPCR)

Total RNA was extracted from cauda equina and sciatic nerves of healthy control rats and rats subjected to EAN at day 17 using the QIAGEN RNEAsy kit. Extracted RNA was reverse transcribed into cDNA, using Taqman Reverse Transcription reagents (Applied Biosystems). qPCRs were performed on an Applied Biosystem ABI7700 sequence detection system and analyzed using the comparative Ct method. Sequences of primers used were as follows: rat 18S, forward 5’-GCTGGAATTACCGCGGCT-3’, reverse 5’-CGGCTACCACATCCAAGGAA-3’; p75^NTR^, forward 5’-AAGGGGCGGGGCATTGTGGTA-3’, reverse 5’-GTTGGGTAGGGGCCTGGAAGTGGT-3’. Data are relative to 18S then normalized against the healthy control.

### Western blot analysis

To assess myelin protein expression, total protein lysates were prepared from cauda equina and sciatic nerves from EAN rats, separated by SDS-PAGE and transferred to PVDF membrane. Membranes were probed with an antibody against myelin basic protein (MBP; AB980, Millipore Bioscience Research Reagents), a myelin marker, and actin as a loading control. Each blot shown in the figure is a representative of at least three independent experiments. Quantification of the Western blot bands was achieved by scanning the blots using a FujiFilm LAS-3000 (FujiFilm). The optical density value for each band was determined using NIH ImageJ, corrected to a loading control, and then normalized against the vehicle control conditions.

### Animal immunohistochemistry

EAN animals (rats and mice) were perfused transcardially with 4% PFA, cauda equina and/or sciatic nerves isolated, frozen, and 10 μm cryosections separated by at least 80 μm collected. The cauda equina or sciatic nerve sections were immunostained with the myelin marker MBP (AB980, Millipore Bioscience Research Reagents), amyloid precursor protein (APP; S12700, Invitrogen), pan voltage-gated Sodium channel (pan-Nav, S8809, Sigma), contactin-associated protein 1 (Caspr, a generous gift from Professor Elior Peles, Weizmann Institute of Science, Israel; Gordon et al., 2014), βIII-tubulin (G7121, Promega), IBa1 (WAKO, 019-19741), or p75^NTR^ (G323A, Promega), followed by the appropriate fluorophore-conjugated secondary antibodies (Invitrogen). The sections were then washed in PBS, and mounted in DAKO mounting medium with DAPI. Six sections per animal from a minimum of three animals per group were analyzed, and images captured by confocal microscopy. The proportion of labeled cells per section was calculated for each animal, and averaged across groups.

### Transmission EM

Rats were perfused with 4% PFA, cauda equina dissected, postfixed overnight at 4°C in Karnoversusky’s fixative, and subsequently processed for resin embedding, as previously described (Xiao et al., 2011, 2013). Semi-thin sections (0.5 μm) were cut and stained with toluidine blue and bright field image taken at 100× magnification. Representative samples were then chosen, and ultrathin sections (90 nm) were cut. EM images were captured using a Siemens Stereoskop Transmission Electron Microscope (Siemens) at 3000× magnification. For quantification, three to four animals per treatment group were used, and minimum six images per animal were taken randomly and analyzed covering the majority of the nerve sections.

### Spectral reflectance confocal microscopy imaging

Spectral reflectance confocal microscopy (SCoRe) imaging was performed on sciatic nerve sections to assess the extent of myelin damage in EAN mice using published methods (Schain et al., 2014). Briefly, mice were perfused with 4% PFA, sciatic nerves dissected (2 mm proximal to the bifurcation of the common peroneal and tibial division), frozen and cryosectioned at 12 μm. Longitudinal sections of sciatic nerves were imaged via a Zeiss 780 Meta confocal microscope with a water immersion objective (Zeiss W Plan-Apochromat 20×/1.0 NA DIC M27 70mm) using 458, 561, and 633-nm laser wavelength through the Tunable Lazer In Tune 488–640 filter/splitter wheel and a 20/80 partially reflective mirror. The reflected light was collected using three photodetectors set to collect light through narrow bands defined by prism and mirror sliders, centered around the laser wavelengths 488, 561, and 633 nm. The channels from each photodetector were then additively combined as a one color composite. All images were acquired using the same settings, analyzed by an operator blinded to conditions using NIH Fiji (ImageJ) software with the threshold function and a minimum cutoff for positive pixels set at 50. The percentage area of positive signal was computed for each image. For quantification, a minimum three tile scans (three images per tile, using a 20×/1.0NA objective at a z-depth 4 μm from the tissue surface) per treatment group were used and statistically analyzed.

### Functional running task assessment

To assess functional outcomes following cyclo-dPAKKR treatment in EAN, animals were videoed on a transparent treadmill using the DigiGait 8 program system (eMouse Specifics) as previously described (Gonsalvez et al., 2017). A simple running task was designed, in which mice were set to complete a 36-s run at a treadmill speed of 15 cm/s. Mice that could not maintain running for the 36-s interval were deemed to fail, as described previously (Gonsalvez et al., 2017). During the running task, video was captured and the running time before failure was analyzed using the DigiGait 8 program. Mice were tested with the running task every 3 d. It typically took 15- to 20-s running time for mice to successfully complete 10 continuous uninterrupted steps as previously described (Gonsalvez et al., 2017).

### Statistical analysis

All data are presented as mean ± SEM, or mean ± 95% confidence interval. Differences between pairs of groups for clinical scores were tested by Mann–Whitney *U* test. Repeated measures two-way ANOVA testing was used to determine the interaction between groups and time, and genotypes, in all cases significance was set at *p* < 0.05. All statistical testing was performed using GraphPad Prism 6 software.

## Results

### Expression of p75^NTR^ is increased in EAN nerves

**Figure 1.**
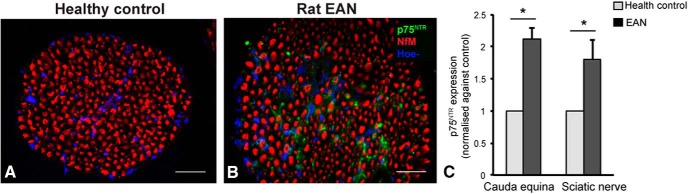
P75^NTR^ expression is increased in injured nerves of rat EAN. ***A***, ***B***, Representative immunohistochemical images of p75^NTR^ and neurofilament (NfM) staining on transverse sections of cauda equine from healthy adult (control, ***A***) and EAN rats (***B***). Immunoreactivity for p75^NTR^ protein is substantially greater in cauda equina sections of EAN rats than healthy controls. Scale bar, 20 μm. ***C***, p75^NTR^ mRNA expression in cauda equina and sciatic nerves of health and EAN rats via qPCR analysis. P75^NTR^ mRAN is significantly upregulated in both cauda equina and sciatic nerves of EAN rats than healthy controls (data: mean ± SEM, relative to 18S then normalized against the healthy control, two-way repeated measures ANOVA *p < 0.01, *n* = 3–4/group).

to investigate whether targeting p75^NTR^ is a potential therapeutic strategy for treating peripheral demyelinating diseases, we sought to first determine the level of p75^NTR^ expression in injured peripheral nerves of rats subjected to EAN at disease peak (day 17) and age-matched health control (non EAN) rats (Fig. 1). We found a low level of p75^NTR^ expression detected in the cauda equina of healthy adult rats (Fig. 1*A*), whereas abundant p75^NTR^ expression was observed in the cauda equina of rats subjected to EAN (Fig. 1*B*). Further, qPCR analysis revealed a significant increase in p75^NTR^ mRNA expression in the cauda equina and sciatic nerves of rats subjected to EAN compared with age-matched healthy control rats (Fig. 1*C*, *n* = 3–4/group, *p < 0.05). Thus, our results demonstrate that p75^NTR^ expression (both mRNA and protein) is increased in the effected peripheral nerves of rats subjected to EAN.

### Cyclo-dPAKKR ameliorates EAN disease severity in a dose-dependent manner

**Figure 2.**
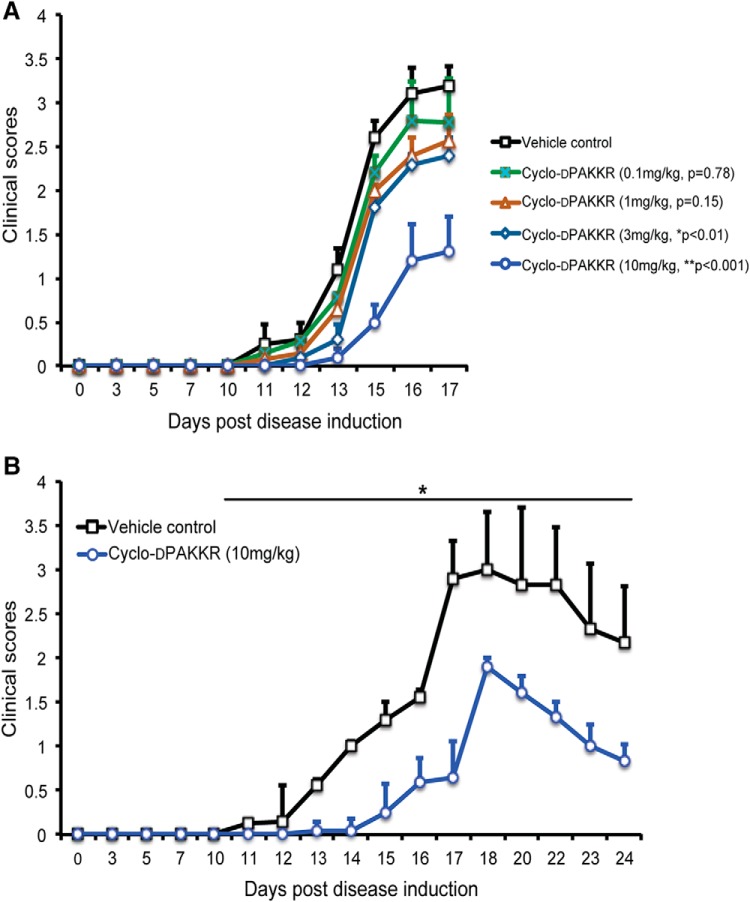
Cyclo-dPAKKR ameliorates EAN in a dose-dependent manner. Time course of disease severity in rats subjected to EAN following daily administration of either vehicle control or Cyclo-dPAKKR from day 1 (data: mean ± SEM, *n* = 6–7/group, Mann–Whitney *U* test). ***A***, Cyclo-dPAKKR reduced disease severity in rat EAN in a dose-dependent manner (3 mg/kg group: *p < 0.01, 10 mg/kg group: **p < 0.001). ***B***, EAN rats treated with cyclo-dPAKKR beyond the disease peak (day 17) exhibited a significantly faster recovery compared with vehicle controls (*p < 0.001).

To investigate whether targeting p75^NTR^ via cyclo-dPAKKR exerts any beneficial influence on EAN, rats received daily intraperitoneal administration of cyclo-dPAKKR over four orders of magnitude (0.1, 1, 3, and 10 mg/kg) or vehicle control (saline) from day 1 (1 day after disease induction) for 17 d. Our data show that cyclo-dPAKKR administration significantly ameliorated EAN disease severity at the two higher doses (3 mg/kg: *p < 0.01 and 10 mg/kg: **p < 0.001), but not the two lower doses (0.1 mg/kg: *p* = 0.78 and 1 mg/kg: *p* = 0.15), compared with the vehicle control (Fig. 2*A*). In addition, animals treated with cyclo-dPAKKR at 10 mg/kg, but not 3 mg/kg, displayed a significant delay (∼3 d) in the onset of EAN disease compared with the vehicle control group (Fig. 2*A*, *p < 0.05).

EAN disease in rats is a monophasic disease that exhibits a stereotypical progression. Following the disease peak at approximately day 17, animals usually undergo a limited degree of spontaneous recovery with a reduction in disease severity (Tran et al., 2010). We next sought to determine if the administration of cyclo-dPAKKR beyond the peak of disease (day 17) ameliorates disease progression and promotes recovery. To do this, EAN was induced in a separate cohort of rats that received daily intraperitoneal administration of cyclo-dPAKKR (10 mg/kg) or vehicle from day 1 for 24 d (Fig. 2*B*). Concordant with the data above (Fig. 2*A*), cyclo-dPAKKR administration significantly delayed the disease onset (∼3 d) compared with vehicle-treated control rats (Fig. 2*B*, *p < 0.05). Delivery of cyclo-dPAKKR beyond the peak of disease continued to ameliorate EAN disease severity compared with the vehicle-treated control group, with animals treated with cyclo-dPAKKR displaying a more rapid reduction in disease scores during the recovery phase compared with vehicle-treated control animals (Fig. 2*B*, *n* = 6–7 mice/group, *p < 0.01), suggesting that treatment with cyclo-dPAKKR beyond disease peak accelerates recovery in EAN.

### Cyclo-dPAKKR abrogates myelin damage in EAN

**Figure 3.**
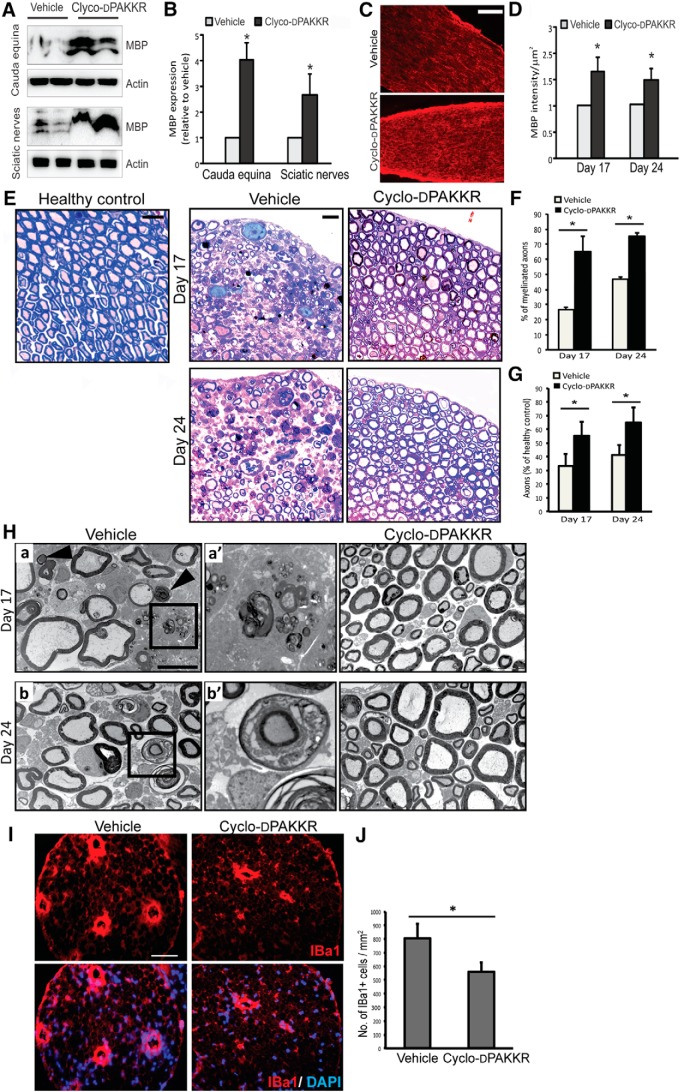
Cyclo-dPAKKR limits the extent of myelin and axonal pathology in EAN. Analyses of cauda equina or sciatic nerves isolated from age-matched healthy control (no EAN) or rats subjected to EAN at day 17 or day 24 after daily treatment with Cyclo-dPAKKR (10 mg/kg) or vehicle control (data: mean ± SEM, *n* = 3–4/group/time point, two-way repeated measures ANOVA *p < 0.05). ***A***, ***B***, Western blot (***A***) and densitometric analyses (***B***) of Western blot bands from cauda equina and sciatic nerve lysates of EAN rats at day 17. ***C***, ***D***, Immunohistochemistry against the myelin marker MBP (***C***) and quantitation of MBP intensity (***D***) in cauda equina sections from EAN rats. Scale bar, 50 μm. ***E***, Representative toluidine blue staining images of cauda equine. Scale bar, 10 μm. ***F***, Quantification of the percentage of myelinated axons in cauda equine. There are significantly more myelinated axons in animals treated with Cyclo-dPAKKR than vehicle controls. ***G***, Quantification of the axonal density in cauda equina. Axonal density in EAN rats is expressed as a percentage of axonal density in health control rats. Axonal density in vehicle-treated animals was significantly reduced by almost 60–70% (30–40% of healthy control), whereas Cyclo-dPAKKR administration protected the axonal loss (∼60% of healthy control). ***H***, Representative electron micrographs of myelinated axons in cauda equina. Scale bar, 2 μm. Panel ***a’*** (a higher magnification image from panel ***a***) shows macrophage-mediated demyelination, as evident by the last vestige of compact myelin being stripped from an axon by a macrophage filled with compact myelin debris. Panel ***b’*** (a higher magnification image from panel ***b***) shows the normal appearing myelin sheath penetrated with macrophages (arrows indicate myelin fragments). ***I***, Representative immunohistochemical images of IBa1 (a marker for macrophages) in transvers sections of cauda equina of rats subjected to EAN at disease peak (day 17). Scale bar, 50 μm. ***J***, Quantification of IBa1+ macrophages from (***F***) reveals that Cyclo-dPAKKR significantly reduced macrophage infiltration in injured nerves compared with vehicle controls.

Demyelination and axonal degeneration are key pathologic features of rodents with EAN and patients with demyelinating peripheral neuropathy, and ultimately influences disease prognosis (Nagappan and Lu, 2005). Having shown that cyclo-dPAKKR ameliorates the clinical deficits in rat EAN (Fig. 2), we next sought to determine the neuropathological basis for its beneficial effects. We first measured expression of the integral myelin protein MBP in injured peripheral nerves via Western blot and immunohistochemical analyses (Fig. 3*A–D*). Both sciatic nerves and cauda equina were dissected at days 17 and 24, followed by total protein extraction and probing with an antibody directed against MBP. We found significantly more MBP expression in both peripheral nerves of EAN rats treated with cyclo-dPAKKR at both time points compared with vehicle-treated controls (Fig. 3*A*,*B*, *p < 0.05). This result was confirmed by assessing MBP protein expression on cauda equina sections using immunohistochemistry (Fig. 3*C*,*D*, *p < 0.05). Collectively, these data suggest that cyclo-dPAKKR administration protects against the loss of myelin protein expression in injured peripheral nerves of EAN animals *in vivo*.

To further determine the extent of myelin pathology in these animals, we undertook an ultrastructural analysis of myelinated axons via histology and EM (Fig. 3*E–H*). We first assessed the percentage of myelinated axons at two time points (days 17 and 24). Our data revealed significantly more myelinated axons in the cauda equina of rats subjected to EAN and treated with cyclo-dPAKKR compared with vehicle-treated controls at both time points (Fig. 3*E*, quantitated in *F*, *p < 0.05). We also examined the density of axons, calculating the data as a percentage relative to healthy control (no EAN) rats (Fig. 3*E*, quantitated in *G*). We also quantitated axonal numbers and found that the number of axons in vehicle-treated animals was significantly reduced to 30–40% of the healthy control, whereas cyclo-dPAKKR administration protected against axonal loss at disease peak and during recovery (∼60% of healthy control; Fig. 3*G*, *n* = 3–4/group/time point, *p < 0.05), supporting out prior finding that cyclo-dPAKKR treatment reduces the extent of demyelination at the peak of disease and during the recovery phase in EAN (Fig. 3*F*).

To further determine if cyclo-dPAKKR exerts a protective influence on myelin integrity in EAN, we assessed myelin ultrastructure via EM analysis (Fig. 3*H*). Concordant with the bright field images (Fig. 3*E*), we found substantially more myelinated axons in the cauda equina of cyclo-dPAKKR-treated groups compared with vehicle controls at both time points (Fig. 3*H*), further suggesting that cyclo-dPAKKR limits the extent of demyelination. It is well established that in patients with demyelinating peripheral neuropathy and animals with EAN, injured peripheral nerves commonly display patterns of macrophage-mediated demyelination such as phagocytosis of compact myelin lamellae following the displacement of the normal-appearing Schwann cell outer mesaxon from the compact myelin membrane (Carenini et al., 2001; Pollard and Armati, 2011). To determine if cyclo-dPAKKR reduced the extent of inflammatory demyelination, we assessed EM images at a higher-magnification. We found that cyclo-dPAKKR treatment markedly limited the extent of macrophage-mediated demyelination in EAN compared with vehicle-treated animals (Fig. 3*H*). In the cauda equina of vehicle-treated rats, we readily observed compact myelin being stripped from an axon by a macrophage filled with myelin debris (Fig. 3*H*, panels *a*,*a’*, macrophage shown in panel *a’*) and the normal appearing myelin sheath penetrated with macrophages (Fig. 3*H*, panels *b*,*b’*). However, these patterns of macrophage-mediated demyelination were rarely observed in the cauda equina of cyclo-dPAKKR-treated animals (Fig. 3*H*), suggesting a limited extent of inflammatory demyelination. The activation, proliferation and recruitment of phagocytic macrophages are a key innate cellular response that contributes to inflammatory demyelination in EAN (Xia et al., 2010). Having shown limited macrophage-mediated demyelination following cyclo-dPAKKR treatment in EAN (Fig. 3*H*), we next investigated macrophage infiltration in the injured peripheral nerves of these animals and found that cyclo-dPAKKR treatment significantly reduced the number of infiltrating IBa1+ macrophages in cauda equina sections at disease peak (day 17) compared with vehicle-treatment (Fig. 3*I*, quantitated in *J*, *n* = 3–4/group, *p < 0.05), indicating that cyclo-dPAKKR administration influences the extent of macrophage infiltration into affected peripheral nerves in EAN. Together, our data demonstrate that cyclo-dPAKKR exerts a protective effect on myelinated peripheral nerves subjected to EAN, in particular reducing the extent of demyelination.

### Cyclo-dPAKKR reduces the extent of axonal damage in EAN

**Figure 4.**
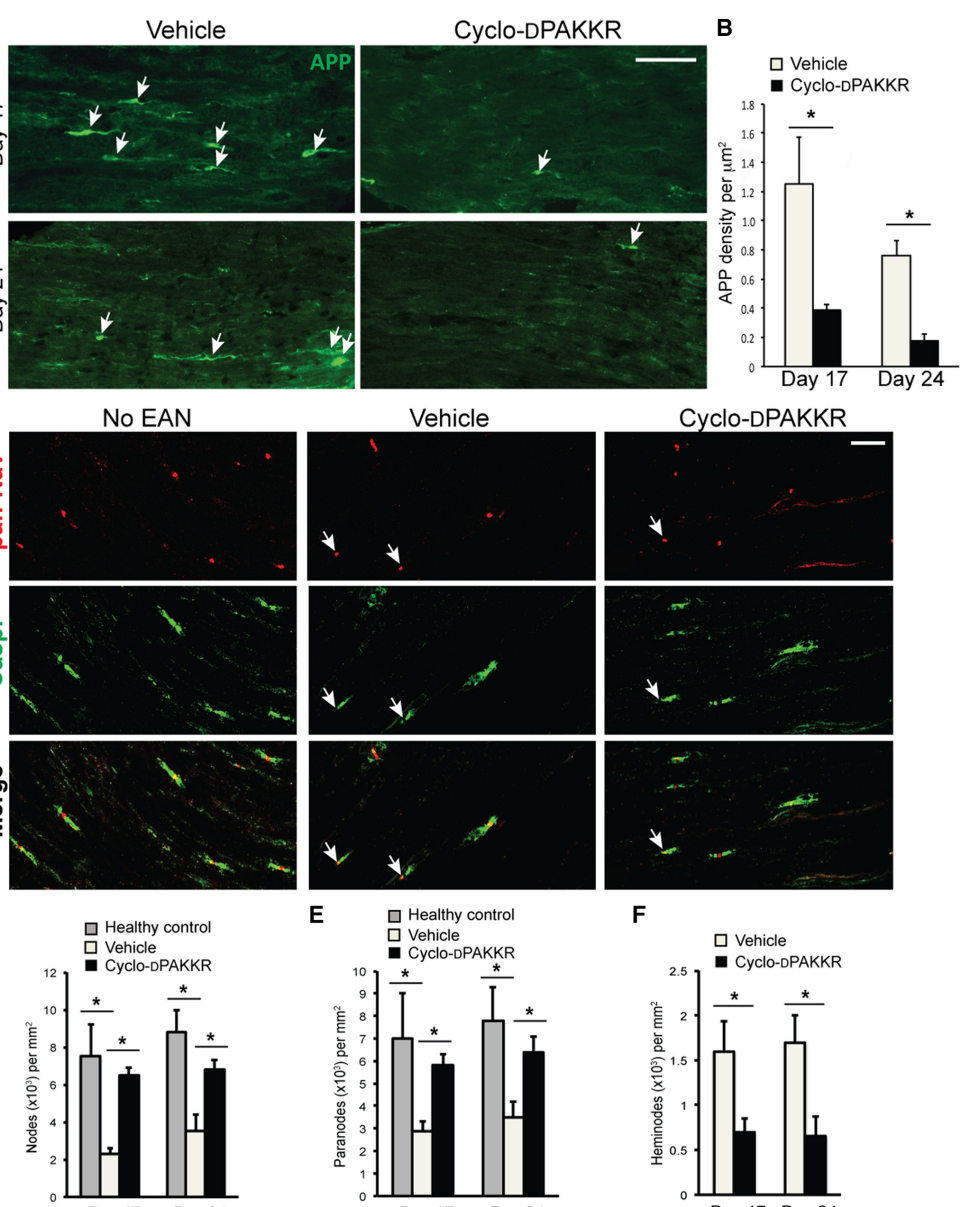
Cyclo-dPAKKR protected against axonal degeneration in EAN. Analyses of cauda equina isolated from rats subjected to EAN at day 17 or day 24 after daily treatment with Cyclo-dPAKKR (10 mg/kg) or vehicle control, or age-matched healthy control rats (no-EAN). ***A***, ***B***, Representative immunohistochemical images of APP (***A***) and quantitation of APP+ axonal spheroids (***B***) in longitudinal sections of cauda equina isolated from rats subjected to EAN. Administration of Cyclo-dPAKKR resulted in significantly fewer APP+ axonal spheroids compared with vehicle controls (data: mean ± SEM, *n* = 3–4/group/time point, two-way repeated measures ANOVA *p < 0.01, scale bar, 20 μm). ***C***, Representative immunohistochemical images of pan-Nav (red) and Caspr (green) expression in longitudinal sections of cauda equina of rats subjected to EAN or health controls. Scale bar, 20 μm, arrows indicate heminodes. ***D***, ***E***, Quantitation of the density of pan-Nav-expressing nodes (***D***) and Caspr-expressing paranodes (***E***) at nodes of Ranvier in the cauda equina of rats subjected to EAN or health controls (data: mean ± SEM, *n* = 3–4/group/time point, *p < 0.05). ***F***, Quantitation of the density of heminodes at nodes of Ranvier from ***C*** showing significantly fewer heminodes in cyclo-dPAKKR-treated animals compared with controls (data: mean ± SEM, *n* = 3–4/group/time point, *p < 0.05).

Increasing evidence suggest that myelin loss ultimately leads to the degeneration of exposed axons, which correlates with the progression of disability in human autoimmune-mediated peripheral demyelinating neuropathies and rodent EAN (Bechtold et al., 2005; Pollard and Armati, 2011; Kieseier et al., 2012). Having shown that cyclo-dPAKKR limits the extent of demyelination in EAN, we next sought to determine if it also limits axonal degeneration. To do this, we first assessed the accumulation of axonal spheroids containing APP, a robust indicator of acute axonal damage to axonal structure (Trapp et al., 1998) in the longitudinal sections of cauda equina of animals subjected to EAN at peak (day 17) and recovery (day 24; Fig. 4*A*,*B*). We found that APP+ axonal spheroids (= axonal spheroids of ∼3–5 μm in diameter) were clearly visible in vehicle-treated animals (Fig.4 *A*, approximately ∼5 per longitudinal section). Animals treated with cyclo-dPAKKR displayed a significant reduction in the accumulation of APP+ axonal spheroids compared with vehicle-treated control animals at both time points (Fig. 4*A*, quantitated in *B*, *n* = 3–4/group/time point, *p < 0.05), suggesting that cyclo-dPAKKR exerts a neuroprotective effect on acute nerve damage in EAN.

It has been shown that disruptions of the nodal and paranodal architecture represent the site of axonal pathology in rodent EAN (Pollard and Armati, 2011) and in patients with chronic inflammatory demyelinating polyneuropathy (CIDP; Cifuentes-Diaz et al., 2011; Doppler et al., 2016). Thus, we investigated the expression of proteins normally clustered at the node of Ranvier and paranodes required for fast axonal conduction (Vabnick and Shrager, 1998). Longitudinal sections of the cauda equina were immunolabeled for voltage-gated pan-sodium channels (pan-Nav) and Caspr, proteins normally localized to the node and paranode, respectively (Arroyo et al., 1999; Gordon et al., 2014; Fig. 4*C*), and quantified nodal and paranodal densities (Fig. 4*D*,*E*). In the vehicle-treated group, we found significantly fewer pan-Nav-expressing nodes (Fig. 4*D*, *p < 0.05) and Caspr-expressing paranodes (Fig. 4*E*, *p < 0.05) compared with healthy (no EAN) control animals. However, these changes of nodal architecture in EAN are rescued by the treatment of cyclo-dPAKKR. We found cyclo-dPAKKR administration resulted in significantly more pan-Nav-expressing nodes (Fig. 4*D*, *p < 0.05) and Caspr-expressing paranodes (Fig. 4*E*, *p < 0.05) compared with vehicle-treated animals at both time points, suggesting a protective effect on nodal structure. We also observed that asymmetrical nodes and heminodes (Fig. 4*C*, arrows), an indicator of structural damage to nodes and paranodal demyelination in CIDP nerves (Cifuentes-Diaz et al., 2011), were visible in animals subjected to EAN but not in healthy control animals (data not shown). Quantification of the density of heminodes revealed significantly fewer heminodes in the nerves of cyclo-dPAKKR-treated animals compared with vehicle controls (Fig. 4*F*, *p < 0.05), further suggesting that cyclo-dPAKKR protects the architecture of nodes and paranodes in EAN. Together, our data demonstrate that cyclo-dPAKKR reduces the extent of demyelination and protects against axonal damage in EAN.

### Cyclo-dPAKKR exerts p75^NTR^-dependent therapeutic effects in EAN

**Figure 5.**
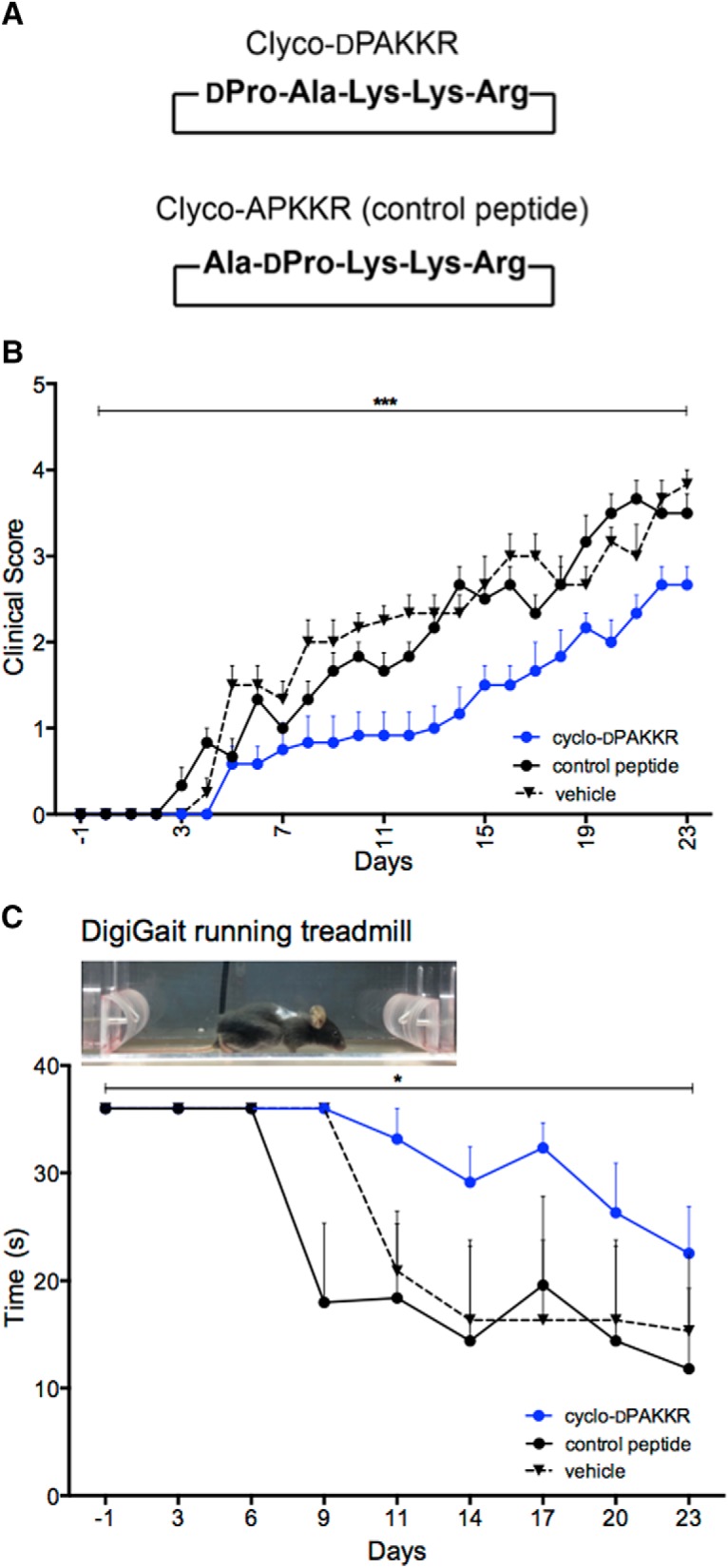
Conformational constraint in Cyclo-dPAKKR determines its therapeutic effect on EAN. ***A***, The sequence structure of the cyclic pentapeptide Cyclo-dPAKKR and its control peptide (cyclo-AdPKKR). Cyclo-dPAKKR is a circular peptide containing a tripeptide motif (KKR) from BDNF shown to bind to p75^NTR^. Cyclo-AdPKKR (control peptide) is also a cyclic pentapeptide containing the same p75^NTR^-binding motif as Cyclo-dPAKKR, but with altered position of Ala and DPro. The five amino acid residues for both peptides are indicated. ***B***, Time courses of EAN disease severity following daily intraperitoneal delivery of either vehicle, cyclo-dPAKKR (10 mg/kg/d) or control peptide (cyclo-AdPKKR, 10 mg/kg/d) in mice after disease induction from day 1. Clinical scores: 0, normal; 1, less lively; 2, mild tail and hindlimb paresis; 3, mild ataxia and limb paresis; 4, severe ataxia and limb paresis; and 5, limb paralysis. Cyclo-dPAKKR, but not control peptide, significantly delayed the onset of disease and reduced EAN severity compared with control peptide (data: mean ± SEM, two-way repeated measures ANOVA ****p < 0.0001, *n* = 6 mice/group). ***C***, Running time before failure in a DidgiGait treadmill running task at moderate speed (15 cm s^−1^). Cyclo-dPAKKR significantly prolonged running capacity in EAN compared with control peptide or vehicle control (data: mean ± SEM, two-way repeated measures ANOVA *p < 0.05, *n* = 6 mice/group).

The tripeptide ‘KKR’ motif is the region of BDNF that binds to p75^NTR^ (Rydén et al., 1995). The head to tail DPro-Ala cyclisation of cyclo-dPAKKR presents the ‘KKR’ motif in the same structural conformation as BDNF (Fletcher et al., 2008). To determine if the beneficial effects that cyclo-dPAKKR exert on EAN is p75^NTR^-dependent, we synthesized a control cyclised peptide (cyclo-AdPKKR) containing the same ‘KKR’ p75^NTR^ binding motif of cyclo-dPAKKR, but in a different conformation as it is contained by a different linker (Ala-LPro; Fig. 5*A*). We have previously shown that this altered conformational state can abolish the trophic effect that cyclo-dPAKKR exerts on peripheral neurons *in vitro* (Fletcher et al., 2008).

In this study, both cyclo-dPAKKR and the cyclised control peptide cyclo-AdPKKR were administered to mice subjected to EAN via daily intraperitoneal administration from day 1 for 23 d, and disease progression assessed by daily clinical scoring and DigiGait functional analysis (Fig. 5*B*,*C*). Concordant with the rat EAN data (Fig. 2), administration of cyclo-dPAKKR in murine EAN significantly ameliorated disease severity and delayed the onset of disease (∼2 d) compared with the vehicle-treated control mice (Fig. 5*B*, *n* = 6 mice/group, ****p < 0.0001). In contrast, the structural control peptide cyclo-AdPKKR exerted no significant effect compared with vehicle-treated controls (Fig. 5*B*, *n* = 6 mice/group, p = 0.477). The finding that cyclo-dPAKKR, but not cyclo-AdPKKR, ameliorates EAN disease severity is also supported by the functional running task assessed by DigiGait analysis. All EAN mice were subjected to a regular running task assessment every 3 d and the running time before failure were analyzed (Fig. 5*C*). We found both the vehicle and control peptide-treated mice began to display a significant drop in their running time from day 9 to 11, whereas the running time of cyclo-dPAKKR-treated mice did not show a significant drop until approximately day 20 (Fig. 5*C*, *p < 0.05, *n* = 6 mice/group), suggesting an ameliorated motor deficit. Moreover, cyclo-dPAKKR-treated mice displayed a significantly prolonged running time compared with the vehicle control group from day 9 onwards (Fig. 5*C*, *p < 0.05, *n* = 6 mice/group), further suggesting better motor performance. In contrast, the control peptide exerted no significant influence on running capacity compared with the vehicle control (Fig. 5*C*). Collectively, these results suggest that the conformational constraint component within cyclo-dPAKKR itself determines its therapeutic effect by facilitating its interaction with p75^NTR^. Our findings that the control peptide cyclo-AdPKKR failed to influence the disease course and motor capacity of mice subjected to EAN indicate that the therapeutic effect of cyclo-dPAKKR is not due to an immune-mediated effect possibly caused by treatment with a peptide.

**Figure 6.**
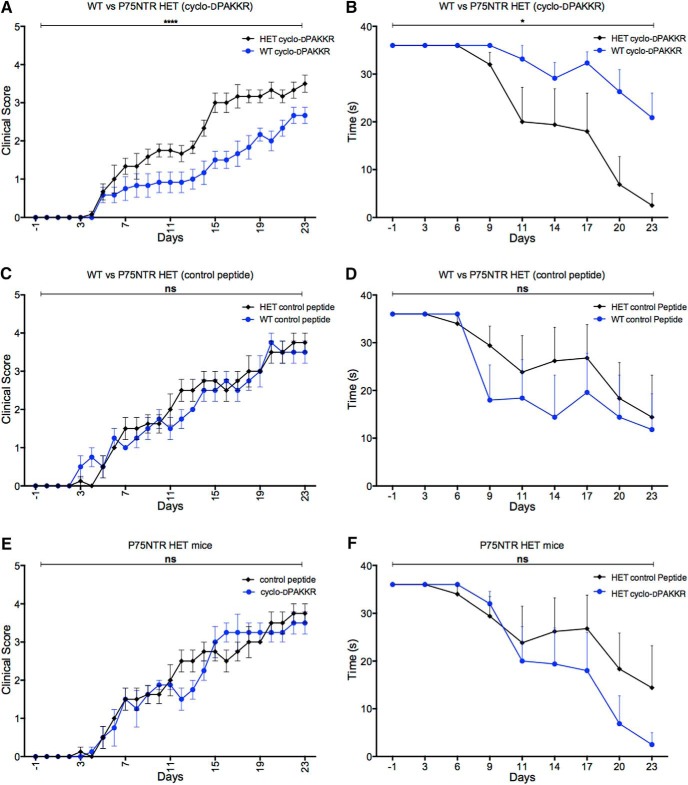
Cyclo-dPAKKR ameliorates EAN in a p75^NTR^-dependent manner. ***A***, ***C***, ***E***, Time courses of EAN disease severity following daily intraperitoneal delivery of either cyclo-dPAKKR (10 mg/kg/d) or control peptide (cyclo-AdPKKR, 10 mg/kg/d) in p75^NTR^ HET or WT littermate control mice after disease induction from day 1 (*n* = 5–6 mice/genotype/group). ***B***, ***D***, ***F***, Running times before failure in a DidgiGait treadmill running task (speed, 15 cm s^−1^). ***A***, ***B***, Cyclo-dPAKKR significantly reduced mean clinical scores (***A***) and improved running capacity (***B***) in WT mice compared with HET mice (data: mean ± SEM, two-way ANOVA with repeated measures, ****p < 0.0001 and *p < 0.05). ***C***, ***D***, No significant difference in clinical scores (***C***) and running times (***D***) between WT and HET mice treated with the control peptide (cyclo-AdPKKR). ***E***, ***F***, No significant difference in clinical scores (***E***) and running times (***F***) between for HET mice treated wither with cyclo-dPAKKR or control peptide.

We further went to determine if cyclo-dPAKKR exerted its effects in a p75^NTR^-dependent manner using p75^NTR^ HET mice. As p75^NTR^ KO (−/−) mice display a developmental loss of >50% sensory neurons in the PNS (Lee et al., 1992; Murray et al., 1999) potentially confounding interpretation in this study, p75^NTR^ HET mice were used. P75^NTR^ HET and WT littermate control mice were induced with EAN, and either untreated, or treated with cyclo-dPAKKR or cyclo-AdPKKR (the control peptide) daily from day 1 for 23 d. Daily clinical scoring revealed no significant difference between disease progression in the untreated p75^NTR^ HET and control WT mice (data not shown), suggesting that p75^NTR^ haploinsufficiency does not influence EAN severity and progression. We found that the clinical scores of p75^NTR^ HET mice treated with cyclo-dPAKKR were significantly higher compared with cyclo-dPAKKR-treated WT mice (Fig. 6*A*, *n* = 6 mice/genotype/group, ****p < 0.0001), suggesting that p75^NTR^ haloinsufficiency attenuates cyclo-dPAKKR’s therapeutic effect. In contrast, the control peptide (cyclo-AdPKKR) exerted no significant effect on disease course between genotypes (Fig. 6*C*, *n* = 6 mice/genotype/group). These data further support the perspective that the conformational constraint component within cyclo-dPAKKR itself is critical for this effect, which is consistent with the data shown in Figure 5*B*. Moreover, there was no significant difference in disease severity between cyclo-dPAKKR and control peptide treatment in p75^NTR^ HET (Fig. 6*E*, *n* = 6 mice/genotype/group). These clinical scores data are supported by the functional motor performance assessed by Digi-gait analysis (Fig. 6*B*,*D*,*F*). We found that WT mice treated with cyclo-dPAKKR performed significantly better in a running task compared with cyclo-dPAKKR-treated HET mice, as evidenced by a significantly prolonged running time (Fig. 6*B*, *n* = 6 mice/genotype/group, *p < 0.05). In contrast, the control peptide (cyclo-AdPAKKR) exerted no significant influence on running capacity between genotypes (Fig. 6*D*, *n* = 6 mice/genotype/group). In addition, there is no significant difference in running times between cyclo-dPAKKR or control peptide-treated p75^NTR^ HET mice (Fig. 6*F*, *n* = 6 mice/genotype/group). Together, our results demonstrate that p75^NTR^ haloinsufficiency attenuates cyclo-dPAKKR’s beneficial effect on ameliorating clinical and functional deficits induced by EAN, suggesting that p75^NTR^ expression is necessary for the therapeutic influence that cyclo-dPAKKR exerts on EAN.

### Cyclo-dPAKKR exerts a p75^NTR^-dependent effect on neuropathology in EAN

**Figure 7.**
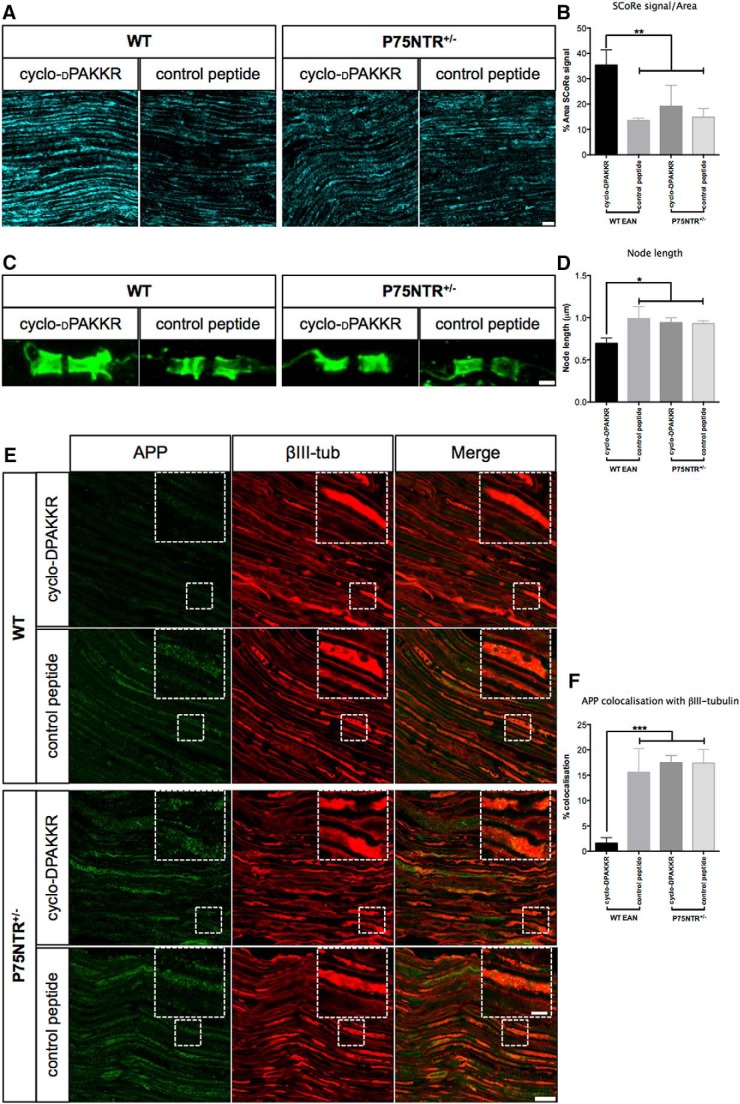
Cyclo-dPAKKR reduces demyelination and axonal degeneration in a p75^NTR^-dependent manner. Histologic analyses of longitudinal sciatic nerve sections of EAN mice following daily treatment of either cyclo-dPAKKR (10 mg/kg) or control peptide (cyclo-AdPAKKR, 10 mg/kg) in p75^NTR^ HET or WT littermate control mice from day 1. All tissues were collected at day 23 (disease peak; data: mean ±SEM, 95% confidence interval, ANOVA with Tukey’s *post hoc* testing *n* = 3 mice/genotype/group, *p < 0.05, **p < 0.01, ***p < 0.001). ***A*, *B***, Representative SCoRe images (***A***) and quantification (***B***) of SCoRe signal as a percentage of total area measured. WT mice treated with cyclo-dPAKKR display a greater percentage area of SCoRe reflected signal than control peptide, indicating more myelinated axons. Cyclo-dPAKKR exerts no significant influence on HET mice compared with control peptide (scale bar, 200 μm). ***C***, ***D***, Representative images (***C***) of Caspr immunostaining and quantification (***D***) of node distance between adjacent Caspr+ paranodes. WT mice treated with cyclo-dPAKKR demonstrate significantly shorter mean node distance compared with mice treated with control peptide. Cyclo-dPAKKR exerts no significant influence on HET mice compared with control peptide (scale bar, 1 μm, minimum 70 nodes per mouse). ***E***, ***F***, Representative images (***E***) of APP and βIII-tubulin double immunostaining and quantitation (***F***) of the percentage of APP+ axonal area colocalizing with βII-tubulin. Cyclo-dPAKKR treatment significantly reduced the percentage of APP+ colocalization with βII-tubulin compared with the control peptide in WT, but not in HET mice (scale bar, 5 μm; scale in inset, 20 μm).

To identify neuropathological evidence of a p75^NTR^-dependent therapeutic effect of cyclo-dPAKKR in EAN, we next assessed the extent of demyelination and axonal degeneration via histology. It has recently been demonstrated that myelin has unique physical properties making it the only structure within neural tissues capable of reflecting light (Schain et al., 2014). To take advantage of the high refractive index of lipid-rich compact myelin, the SCoRe imaging method has been recently developed to assess myelinated axons *in vivo* in both healthy and pathologic conditions (Schain et al., 2014). Here, we examined the extent of myelin damage on longitudinal sections of sciatic nerve from mice subjected to EAN using SCoRe imaging (Fig. 7*A*,*B*). We found that WT mice treated with cyclo-dPAKKR demonstrate a significantly higher percentage of reflected signal compared with mice treated with the control peptide (Fig. 7*A*, quantitated in *B*, *p < 0.05), indicating a greater percentage of compact myelin per area assessed. Concordant with the clinical and functional assessments (Fig. 6), cyclo-dPAKKR exerted no significant influence on SCoRe signal in p75^NTR^ HET mice compared with the control peptide (Fig. 7*A*,*B*), indicating that cyclo-dPAKKR requires the expression of p75^NTR^ to confer beneficial influence on myelin in EAN.

Widening of the node of Ranvier, an indicator of demyelination and axonal damage, is one of the pathologic features commonly observed in peripheral demyelinating neuropathy (Griffin et al., 1996). Next we examined if cyclo-dPAKKR treatment can influence nodal widening in EAN mice via measuring the distance between the paired Caspr+ paranodes in longitudinal sciatic nerve sections of mice treated with cyclo-dPAKKR or control peptide (Fig. 7*C*, quantitated in *D*). Caspr+ heminodes were excluded from this analysis. We found that the node length is significantly shorter in WT mice treated with cyclo-dPAKKR compared with those treated with control peptide (Fig. 7*D*, *p < 0.05), suggesting that cyclo-dPAKKR- protects against nodal widening. Moreover, cyclo-dPAKKR failed to influence nodal length widening in p75^NTR^ HET mice (Fig. 7*C*,*D*). There was no significant difference in the nodal length between WT and p75^NTR^ HET mice treated with the control peptide (Fig. 7*C*,*D*). Collectively, our data suggest that cyclo-dPAKKR requires the normal expression of p75^NTR^ to exert its beneficial influence on nodal structures and axonal damage in EAN.

We further investigated whether cyclo-dPAKKR exerts p75^NTR^-dependent effects on axonal degeneration via assessing the accumulation of APP in mice subjected to EAN (Fig. 7*E*,*F*). APP+ axonal spheroids were readily visible along injured peripheral nerves in the rat model of EAN (Fig. 4*A*); however, only APP+ axonal segments, but not axonal spheroids, were observed in the murine model of EAN (Fig. 7*E*), suggesting the extent of overall axonal degeneration is less severe in the murine EAN model compared with the rat model. As such we decided to analyze the percentage of APP+ immunostaining that was colocalized with the neuronal marker βIII-tubulin (a mask was created using the βIII-tubulin signal and the percentage area of positive APP+ signal was computed; Fig.7*E*, quantitated in *F*). We found that in WT mice, cyclo-dPAKKR treatment significantly reduced the percentage of APP+/βII-tubulin+ colocalization compared with the control peptide group (Fig. 7*E*, quantitated in *F*, *n* = 3 mice/genotype/group, ***p < 0.001), supporting our data observed in rat EAN (Fig. 4*A*). Concordant with clinical, functional and myelin assessments (Figs. 6 and 7*A-D*), the protective effect that cyclo-dPAKKR exerts against axonal degeneration is abolished in p75^NTR^ HET mice, in which cyclo-dPAKKR exerted no significant influence on the percentage of APP+ axonal area compared with the control peptide-treated group (Fig. 7*E*,*F*). Moreover, we found that control peptide treatment exerted no significant effect on the percentage of APP+ axonal area between genotypes (Fig. 7*E*,*F*). Together, our data provide strong neuropathological evidence that p75^NTR^ expression is required for the protective effects that cyclo-dPAKKR exerts against myelin and axonal pathology in EAN, indicating that it ameliorates EAN-induced neuropathology in a p75^NTR^-dependent manner.

## Discussion

In this study, we show that a conformationally constrained BDNF-based p75^NTR^ peptide ameliorates functional, axonal and myelin pathologies induced by EAN disease in both rats and mice. Importantly, these beneficial effects are abolished when the genetic dose of the p75^NTR^ is halved. Together, our data suggest that targeting p75^NTR^ is a novel therapeutic strategy that ameliorates demyelination and axonal degeneration induced by peripheral demyelinating diseases.

### Cyclo-dPAKKR limits the extent of functional deficits and neuropathology in EAN

The altered expression of the neurotrophins and p75^NTR^ in experimental models of peripheral nerve injury and in a range of human peripheral neuropathies (Yamamoto et al., 1998) has resulted in speculation that the neurotrophins could be involved in the pathogenesis and repair of these diseases (Pittenger and Vinik, 2003; Anand, 2004; Zhou and Notterpek, 2016). Two recent studies have investigated the potential of using BDNF as a therapeutic strategy to treat peripheral demyelinating neuropathy. Treatment with recombinant BDNF in rat EAN failed to improve neurologic and motor deficits (Felts et al., 2002). Further, transplantation of BDNF-treated Schwann cells also failed to ameliorate rat EAN (Hou et al., 2013). Consistent with these findings, we found that administration of recombinant BDNF failed to promote the myelination of developing peripheral nerves *in vivo* (Xiao et al., 2013), although it is able to enhance Schwann cell myelination *in vitro* (Xiao et al., 2009b). These studies together demonstrate that BDNF itself is not an optimal therapeutic candidate for treating demyelinating diseases. While the precise reason why BDNF exerts no beneficial effect on myelination during development and rodents subjected to EAN *in vivo* remains unclear, it is important to be cognisant of the poor drug-like properties of BDNF and the complexity of BDNF signaling (Chao, 2003), in particular in the context of myelination (Xiao et al., 2009b). In this case, we have identified that targeting of p75^NTR^, a positive regulator of both peripheral neuron survival and myelination (Lee et al., 1992; Cosgaya et al., 2002; Xiao et al., 2009b), could be a new approach that can protect the PNS after injury.

We have previously identified that cyclo-dPAKKR promotes the survival and myelination of peripheral neurons (Fletcher et al., 2008; Fletcher and Hughes, 2009; Xiao et al., 2013). In this study, we have demonstrated that cyclo-dPAKKR administration significantly ameliorated EAN disease severity and accelerated recovery compared with controls. Cyclo-dPAKKR-treated animals displayed significantly better motor performance compared with both vehicle and control peptide-treated groups. Moreover, these functional benefits of cyclo-dPAKKR are strongly supported by the limited extent of neuropathology, as evident by reduced demyelination and axonal damage as well as immune cell infiltration in injured nerves compared with control groups. Importantly, analysis of the node of Ranvier revealed that the loss of Nav-expressing domains, disruption of paranodal structures and widening of the nodal length, as are also observed in human peripheral demyelinating neuropathy (Griffin et al., 1996; Pollard and Armati, 2011), were attenuated in cyclo-dPAKKR-treated animals. This finding is particularly interesting as it suggests that cyclo-dPAKKR treatment exerts protective effects on the axo-glial junction and reduces the extent of paranodal demyelination in EAN. There is an emerging consensus that the progression of disability in human peripheral demyelinating neuropathy and rodent EAN correlates with the accumulation of axonal damage, which in turn is influenced by the extent of demyelination (Bechtold et al., 2005; Pollard and Armati, 2011; Kieseier et al., 2012). Together, our data indicate that cyclo-dPAKKR treatment either reduces the extent of demyelination, or promotes more complete remyelination, with the net effect of reducing axonal damage, providing strong anatomic evidence that supports the improved functional outcomes in EAN.

### Cyclo-dPAKKR’s effects are p75^NTR^ dependent

Both BDNF and cyclo-dPAKKR have previously been shown to promote peripheral nerve myelination via p75^NTR^ (Xiao et al., 2009b, 2013). In this study, our data consistently demonstrate that p75^NTR^ expression is required for the beneficial influence that cyclo-dPAKKR exerts on EAN disease severity, motor function, myelin and axonal pathology. It is interesting that cyclo-dPAKKR failed to ameliorate EAN induced disease in p75^NTR^ HET mice that still express p75^NTR^, but at a reduced level, suggesting that the beneficial effect of cyclo-dPAKKR may require a threshold level of p75^NTR^ expression, which ultimately leads to optimal downstream p75^NTR^ signaling. This speculation can also partially explain our finding that cyclo-dPAKKR exerts a dose-dependent effect on EAN. Nevertheless, our data strongly suggest that cyclo-dPAKKR exerts a therapeutic effect on EAN which is dependent on p75^NTR^ expression. This is further supported by the finding that the cyclised control peptide (cyclo-AdPKKR), that contains the same ‘KKR’ p75^NTR^ binding motif of cyclo-dPAKKR, but in an altered conformational state, exerted no influence on EAN. This result indicates that the beneficial effects that cyclo-dPAKKR exert on EAN are crucially dependent on the proper conformation of the ‘KKR’ p75^NTR^ binding motif. The lack of effect of cyclo-AdPKKR compared with cyclo-dPAKKR reflects our previous observations when examining neuronal survival *in vitro* (Fletcher et al., 2008). We have previously shown that cyclo-dPAKKR adopts a single highly constrained conformation in solution (Fletcher et al., 2008). It is therefore not surprising that even a seemingly modest change in the linker could lead to a dramatic change in conformational preference, sufficient to alter ligand-receptor interactions and subsequent downstream signaling. Ultimately, a greater understanding of the molecular interaction, specificity, binding affinity and stoichiometry between cyclo-dPAKKR and p75^NTR^ is required to definitively identify the molecular basis of the differential effects of cyclo-dPAKKR and cyclo-AdPKKR on EAN.

At a cellular level, it remains interesting to determine the cell type(s) that cyclo-dPAKKR targets to ameliorate EAN. It is well established that p75^NTR^ is expressed in neurons and Schwann cells, and it promotes the viability of subsets of peripheral neurons (Lee et al., 1992; Murray et al., 1999) and influences Schwann cell myelination (Cosgaya et al., 2002; Chan et al., 2006) during development. We and others have shown that p75^NTR^ KO mice display a loss of >50% sensory neurons compared with WT mice (Lee et al., 1992; Murray et al., 1999). It has, however, been uncertain as to whether this effect is mediated via loss of p75^NTR^ signaling in isolation, or whether it is due to the loss of signaling through “high-affinity” neurotrophin receptor complexes (i.e. p75^NTR^ in combination with the Trk receptors). Interestingly, we have previously demonstrated that cyclo-dPAKKR promotes the survival of purified embryonic chick sensory neurons in culture (Fletcher et al., 2008), strongly suggesting that selective p75^NTR^ induced signaling is neurotrophic. In this study, we found that cyclo-dPAKKR administration significantly protected against EAN-induced axonal damage, suggesting a neuroprotective effect. These findings collectively suggest that cyclo-dPAKKR administration in the context of demyelinating neuropathies may not only limit demyelination but also potentially directly ameliorate secondary axonal degeneration and neuronal loss. Further, we have previously identified that cyclo-dPAKKR selectively targets neuronally expressed p75^NTR^ to promote peripheral nerve myelination (Xiao et al., 2013). Thus, it is plausible that cyclo-dPAKKR could directly target neurons to promote both their survival and remyelination, thus effectively ameliorating EAN-induced neuropathology, which ultimately determines disease progression and severity.

Interestingly, macrophages and T cells also express p75^NTR^ (Flügel et al., 2001; Pérez-Piñera et al., 2006). In adition, our data also show that cyclo-dPAKKR reduced immune cell infiltration into affected peripheral nerves, suggesting cyclo-dPAKKR may act by more than one mechanism to ameliorate disease severity in EAN. However, based on clinical scoring, cyclo-dPAKKR-treated EAN animals still become sick, indicating that cyclo-dPAKKR itself does not block antigen presentation or the induction of T-cells. We have shown that neither injection of p75^NTR^ antisense oligonucleotides nor the genetic knockout of p75^NTR^ impedes the induction of experimental autoimmune encephalomyelitis (Soilu-Hanninen et al., 1999), a T-cell-mediated autoimmune disease in the CNS that has similar pathogenesis to EAN in the PNS. Collectively, this suggests that macrophage expression of p75^NTR^ may not play a significant role in EAN induction, but it nevertheless remains unclear whether the observed decrease in infiltrating macrophages is a direct effect on the macrophages themselves, or is an indirect effect secondary to myelin repair.

While the utility of p75^NTR^ heterozygous mice, in which there is a global p75^NTR^ haploinsufficiency, enabled identification of the requirement of p75^NTR^ for cyclo-dPAKKR to exert its therapeutic benefit, it does not enable direct insight into the specific pathologic processes that cyclo-dPAKKR influences. Whether this is reduced axonal damage, enhanced myelin repair or immunomodulation remains unclear. This technical limitation precludes identification of a clear-cut cellular mechanism underlying the beneficial effects that cyclo-dPAKKR exerts on EAN. Thus, future studies are required to determine the precise mechanisms of cyclo-dPAKKR action through adopting inducible genetic approaches in which p75^NTR^ is selectively deleted from peripheral neurons, Schwann cells or immune cells in the adult PNS, without interfering with the normal development of these cells. Future experiments are also required to determine the therapeutic efficacy of cyclo-dPAKKR on EAN under different treatment paradigms, such as commencing treatment after disease symptoms appear.

In summary, we have identified that cyclo-dPAKKR, a structural mimetic of the region of BDNF that binds to p75^NTR^, ameliorates EAN disease severity and functional deficits accompanied by strong neuroprotection against myelin and axonal damage, effects dependent on p75^NTR^ expression. Interestingly, cyclo-dPAKKR itself displays drug-like properties, in that it is of low molecular weight, metabolically stable and readily crosses cell membranes (Fletcher et al., 2008; Fletcher and Hughes, 2009). Demyelination followed by axonal damage is a common and integral component of demyelinating diseases, and our work identifying cyclo-dPAKKR exerts p75^NTR^-dependent neuroprotective effects on EAN provides new insight into the future development of neuroprotective therapeutics for peripheral demyelinating diseases.

## Synthesis

Reviewing Editor: Jun Chen, University of Pittsburgh Decisions are customarily a result of the Reviewing Editor and the peer reviewers coming together and discussing their recommendations until a consensus is reached. When revisions are invited, a fact-based synthesis statement explaining their decision and outlining what is needed to prepare a revision will be listed below.

The revision has addressed my previous concerns. However, some minor errors need to be fixed. For example, the last paragraph was duplicated.
